# Retention on Buprenorphine Is Associated with High Levels of Maximal Viral Suppression among HIV-Infected Opioid Dependent Released Prisoners

**DOI:** 10.1371/journal.pone.0038335

**Published:** 2012-05-31

**Authors:** Sandra A. Springer, Jingjun Qiu, Ali Shabahang Saber-Tehrani, Frederick L. Altice

**Affiliations:** 1 Section of Infectious Disease, AIDS Program, Yale University School of Medicine, New Haven, Connecticut, United States of America; 2 Division of Epidemiology of Microbial Diseases, Yale University School of Public Health, New Haven, Connecticut, United States of America; 3 The Faculty of Medicine, Centre of Excellence on Research in AIDS, University of Malaya, Kuala Lampur, Malaysia; University Hospital Zurich, Switzerland

## Abstract

**Introduction:**

HIV-infected prisoners lose viral suppression within the 12 weeks after release to the community. This prospective study evaluates the use of buprenorphine/naloxone (BPN/NLX) as a method to reduce relapse to opioid use and sustain viral suppression among released HIV-infected prisoners meeting criteria for opioid dependence (OD).

**Methods:**

From 2005–2010, 94 subjects meeting DSM-IV criteria for OD were recruited from a 24-week prospective trial of directly administered antiretroviral therapy (DAART) for released HIV-infected prisoners; 50 (53%) selected BPN/NLX and were eligible to receive it for 6 months; the remaining 44 (47%) selected no BPN/NLX therapy. Maximum viral suppression (MVS), defined as HIV-1 RNA<50 copies/mL, was compared for the BPN/NLX and non-BPN/NLX (N = 44) groups.

**Results:**

The two groups were similar, except the BPN/NLX group was significantly more likely to be Hispanic (56.0% v 20.4%), from Hartford (74.4% v 47.7%) and have higher mean global health quality of life indicator scores (54.18 v 51.40). MVS after 24 weeks of being released was statistically correlated with 24-week retention on BPN/NLX [AOR = 5.37 (1.15, 25.1)], having MVS at the time of prison-release [AOR = 10.5 (3.21, 34.1)] and negatively with being Black [AOR = 0.13 (0.03, 0.68)]. Receiving DAART or methadone did not correlate with MVS.

**Conclusions:**

In recognition that OD is a chronic relapsing disease, strategies that initiate and retain HIV-infected prisoners with OD on BPN/NLX is an important strategy for improving HIV treatment outcomes as a community transition strategy.

## Introduction

Combination antiretroviral therapy (cART) has markedly reduced morbidity and mortality from HIV disease. [1] Despite more simplified regimens and a myriad of interventions to improve HIV detection and treatment, fewer than 20% of all people with HIV/AIDS (PLWHA) in the United States (U.S.) achieve viral suppression (VS). [2] Maximal viral suppression (MVS), defined as achieving a HIV-1 RNA level below 50 copies/mL, is associated with improved HIV treatment outcomes compared to those who suppress HIV-1 RNA levels to less than 400 copies/mL, but who do not achieve MVS. [3] Sustained VS, using effective cART as prevention, has recently been demonstrated to reduce HIV transmission by 96% among HIV serodiscordant heterosexual couples. [4].

Individuals with HIV disease transitioning to the community from the criminal justice system (CJS) are a particularly vulnerable population where effective interventions are urgently needed. [5] Within U.S. prisons, HIV and AIDS is three and four times greater, respectively, than found in the general population. [6] Moreover, one-sixth of all PLWHA in the U.S. pass through the CJS annually, [6] making these settings important sites for detection and treatment of HIV infection. [7] Though PLWHA in prisons achieve high rates of viral suppression [8] while incarcerated and have recently achieved similar mortality as non-prisoners, [9] the transition to the community is associated with high post-release mortality [10] and poor HIV treatment outcomes. [8], [11], [12].

Reasons for poor post-release outcomes among PLWHA are complex and multifactoral, [5] but relapse to alcohol and drug use is common and both are associated with poor HIV treatment persistence and adherence. [13], [14], [15], [16], [17] Pre-incarceration opioid dependence is common, especially among those with HIV infection. [18], [19], [20] The chronic, relapsing nature of opioid dependence results in 85% to 90% of such persons relapsing to opioid use within 1 year after release, regardless of duration of incarceration. [21].

Despite evidence to support successful treatment for opioid dependence using opioid substitution treatment (OST) in community and CJS settings, (e.g. methadone and buprenorphine), [22], [23], [24], [25] OST is seldom available for treatment within the CJS or upon release in the U.S. [26], [27] Cited reasons for the lack of implementation of OST involving CJS populations are multiple, including: 1) ideology that OST is replacing one addiction for another; 2) stringent licensing regulations; 3) concerns about diversion; and 4) costs. [28], [29], [30] In a recently published pilot study of 23 HIV-infected released prisoners with pre-incarceration criteria for opioid dependence, we confirmed that buprenorphine/naloxone (BPN/NLX) treatment was feasible in the vulnerable 12-week post-release period and was highly acceptable and highly associated with short-term viral suppression. [31] We now extend these findings to a larger sample for a longer duration, and explore the impact of BPN/NLX treatment on HIV treatment outcomes compared to similar patients who did not receive BPN/NLX.

## Methods

The rationale and description of the overall prospective study of directly administered antiretroviral therapy (DAART) has been describe [32], [33] as have the procedures for BPN/NLX induction. [31].

### Recruitment

Eligible subjects were recruited during the years 2005–2010 from among 154 HIV-infected prisoners transitioning to the community enrolled in a randomized controlled trial (RCT) of directly administered antiretroviral therapy (DAART). [32], [33], Subjects enrolled in this RCT who met pre-incarceration DSM-IV criteria for opioid-dependence, were assessed for interest in opioid substitution therapy (OST) with either methadone or BPN/NLX. Additional eligibility criteria included: 1) returning to either New Haven or Hartford; 2) age ≥18 years; 3) a negative urine pregnancy test for women and willingness to use contraception; and 4) expressing an interest in MAT. As part of the ongoing parent RCT, subjects were randomized 2∶1 to receive DAART versus self-administered therapy (SAT). [32].

### Study Procedures

Within 90 days before community-release, all subjects underwent informed consent, baseline assessments and chart review. Assessments included demographic information, mental illness and chemical dependence screening using the Mini-International Neuropsychiatric Interview (M.I.N.I), [34] Addiction Severity Index (ASI), [35], [36] and Alcohol Use Disorders Identification Test (AUDIT) [37] and urine toxicology screening. Alcohol and drug use questions referred to the pre-incarceration period to establish historical diagnoses, as no subject was actively using drugs or alcohol while incarcerated based on urine testing. Subjects underwent secondary consent procedures after release to avoid any perceived or real coercion. A more detailed study protocol and baseline characteristics from the parent study have been a recently published. [33] Additional post-release activities included baseline physical exam and weekly assessment of opioid craving (10-point Likert scale), buprenorphine satisfaction (10-point Likert scale), weekly urine toxicology screening using the NIDA-6 (opioids, cocaine, methadone, benzodiazepines, marijuana, methamphetamines) and separate urine tests for oxycodone and buprenorphine (Redwood Biotech, Santa Rosa, CA). Baseline and quarterly HIV-1 RNA levels (Amplicor 1.5; Roche) and CD4 lymphocyte counts (FACS; Quest) were obtained.

### Ethics

The Yale University Human Investigation Committee and the Connecticut Department of Correction Research Advisory Committee approved this study and the parent study is registered at www.clinicaltrials.gov (NCT00786396). Additional assurances to protect the participants were obtained from the Office of Human Research Protection (OHRP) at the Department of Health and Human Services, and a Certificate of Confidentiality from the National Institutes of Health was also obtained. As mentioned above, written consent to participate in the parent trial [33] and this sub-study was obtained from the participants prior to enrollment. If the subject did not want to participate in this sub-study, they were allowed to continue in the parent study. If also at any time they wished to stop the study, they were told they could do so and would be referred to a community substance use treatment program if they wished to continue to receive relapse prevention treatment for opioid dependence.

### Buprenorphine Induction Process

BPN/NLX induction and maintenance procedures have been described previously, [31] and others have more recently described BPN implementation issues in criminal justice settings for patients not infected with HIV. [38] Briefly, BPN/NLX induction was allowed up to 30 days post-release from prison; however, the day of release was targeted when possible. Baseline urine toxicology testing for subjects inducted on the day of release confirmed that there was no relapse that occurred on the day of release or in the immediate 72 hours during incarceration. Due to low expected tolerance at the time of prison-release, subjects were initially administered 2.0 mg/0.5 mg BPN/NLX and increased by 2 mg/0.5 mg increments of BPN/NLX daily, as tolerated, to reduce the craving score to 1, while avoiding opioid agonist side effects. BPN/NLX dose, craving for opioids, opioid withdrawal symptoms, opioid-agonist side effects, and urine toxicology screening were collected daily during the induction and weekly thereafter.

### Buprenorphine Maintenance Therapy (BMT) Procedures

BMT was defined as having received even a single dose of BPN/NLX along with weekly, standardized and manual-based counseling per protocol [39] for 45–60 minutes by a certified substance abuse treatment counselor for the first 12 weeks; frequency of counseling thereafter varied based on clinical response and provider preference. Counseling is considered standard care for patients receiving BPN/NLX. Study personnel linked counseling visits to collection of urine screens and distribution of weekly refill vouchers. The voucher was not contingent on urine specimen results. For those randomized to DAART, BPN/NLX was observed daily along with their cART and other chronically prescribed medications. For those in the SAT arm, a 7-day prescription BPN/NLX voucher was provided to allow the pharmacy to provide the BPN/NLX after providing a urine specimen and attending the weekly counseling sessions for the first 12 weeks of the study.

### Follow-up

Subjects receiving BMT were evaluated daily by the study clinician during the induction phase and at least monthly thereafter. Counselors met with subjects weekly, irrespective of study assignment, and assessed urine toxicology screening, opioid craving, BPN/NLX satisfaction and adverse side effects. Structured interviews and phlebotomy for CD4 lymphocyte count and HIV-1 RNA level were conducted at weeks 4,12 and 24 after release.

### Analytic Strategy

All statistical analyses were conducted using STATA v.10 (StataCorp LP, Texas, USA). Outcomes from the first 24 weeks are reported for all 94 subjects meeting DSM-IV criteria for opioid dependence who were including in the parent RCT. The primary outcome was defined as the proportion of patients achieving maximal virological suppression (MVS), or HIV-1 RNA <50 copies/mL, at 24 weeks. Using logistic regression analyses, univariate analysis was performed to assess the impact of potential independent variables associated with the primary outcome. Subsequently, a multivariate model was fitted to the data, using both backward and forward stepwise regression approaches incorporating Bonferroni correction; *P-*values censored at ≤0.20 were restricted to enter and leave the model. Aikake (AIC) and Bayesian Information Criterion were applied to each model to assess goodness of fit and to avoid over fitting of data. Missing HIV-1 RNA values were considered as failures (HIV-1 RNA >50 copies/mL).

Among the 94 participants who met pre-incarceration DSM-IV criteria for opioid dependence, we sought to conduct a naturalistic study comparing those who initiated buprenorphine maintenance treatment (BMT) with those who did not. Those not initiating BMT included those that selected either methadone maintenance therapy (MMT; N = 9) or no OST (N = 35). Because of the naturalistic nature of the follow-up, those not receiving BMT received what is routinely available as standard of care in community settings for those receiving MMT and no OST (including available counseling).

The independent substance abuse variables of interest were: 24-week retention in BPN/NLX treatment and degree of addiction severity using the Addiction Severity Index (ASI). [35] Urine toxicology results were not collected for the non-BPN/NLX group, therefore they were not included in the univariate or multivariate analysis nor was this group compared to the BMT group. Urine toxicology results were available for the BPN/NLX group only and were measured as the percentage of opioid-free and cocaine-free urine toxicology results over 24 weeks. Missing urine results were adjudicated in the following sequential manner: 1) self-report at weekly visits; and 2) last value carried forward only if a single missing value was noted; 3) for subjects who remained in the trial, missing consecutive urine values were considered positive. Therefore, the proportion of positive urine tests for the BPN/NLX group was calculated as the percent positive out of the number who remained in the trial for each week and included missing value adjudication. Craving and satisfaction scores for the BPN/NLX group only were calculated as the mean for those individuals whose results were reported weekly.

## Results

**Figure 1:**
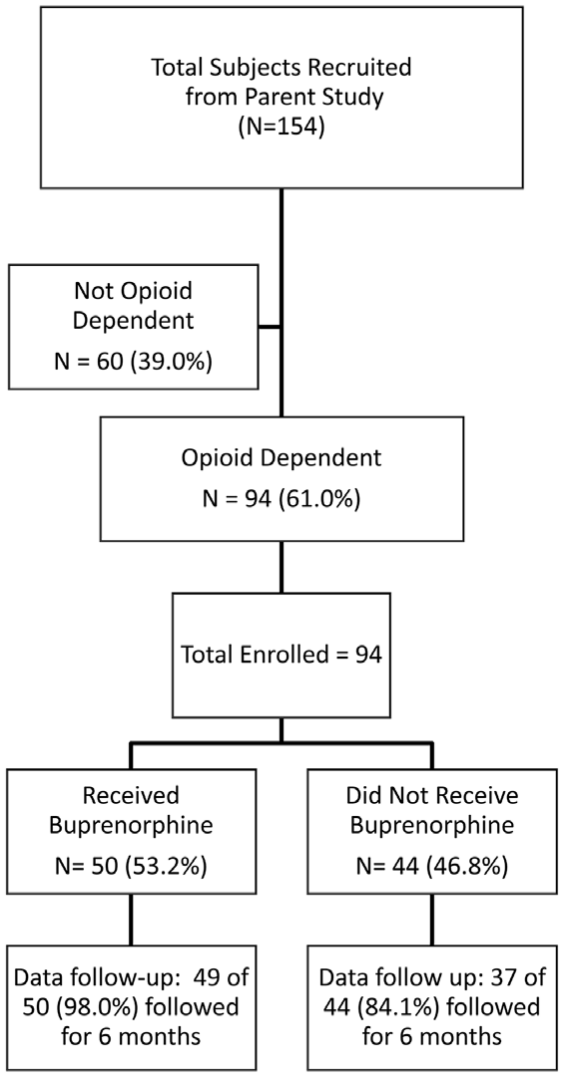
Subject Disposition. Difference of data follow-up between Buprenorphine/Naloxone and Non-Buprenorphine retention is statistically significant, *p* = 0.024.

**Figure 1** depicts the disposition of the 154 subjects who were included in the parent trial: 94 (61%) met pre-incarceration DSM-IV criteria for opioid dependence and about half (N = 50, 53%) opted to receive BPN/NLX (i.e, BPN group). Among the 44 (47%) who chose not to receive BPN/NLX (i.e. Non-BPN group), 9 (20.5%) selected methadone and 79.5% preferred no MAT. The availability of follow-up data for the primary outcomes (HIV-1 RNA levels) at 24 weeks was significantly higher in the BPN group than in the non-BPN group (98% vs. 84.8%, p = 0.024).

**Table 1** compares the baseline characteristics of the two groups (BPN vs Non-BPN). In general, the two groups were similar with regard to age and gender, self-reporting anticipated homelessness at the time of release, and meeting DSM-IV criteria for a major Axis I disorder. Similarly, baseline opioid craving did not differ nor did the levels of addiction severity, cocaine use, depressive symptoms, type of cART regimen prescribed, baseline viral load, CD4 characteristics or proportion that had previously received methadone treatment. The groups were different, however, on two important characteristics; race/ethnicity and city of residence. Hispanics, compared to whites and blacks, were more likely to comprise the BPN group and Hartford site groups.

**Table 1:** Baseline characteristics of study subjects, N (%) for categorical variables and mean±SD for continuous variables.

	BPN/NLX (N = 50)	No BPN/NLX (N = 44)	Total Sample Population (N = 94)	*P* value
Gender				0.10
Male	44 (88.0%)	33 (75.0%)	77 (81.9%)	
Female	6 (12.0%)	11 (25.0%)	17 (18.9%)	
Mean Age (years), S.D.	45.62±6.05	46.59±7.52	46.07±6.76	0.27
Ethnicity				0.00
White	6 (12.0%)	11 (25.0 %)	17 (18.9%)	
Black	16 (32.0%)	24 (54.6%)	40 (42.6%)	
Hispanic	28 (56.0%)	9 (20.4%)	37 (39.4%)	
Study Site				0.01
New Haven	13 (26.0%)	23 (52.3%)	36 (38.3%)	
Hartford	37 (74.0%)	21 (47.7%)	58 (61.7%)	
Homelessness	19 (43.2%)	14 (38.9%)	33 (41.3%)	0.70
Median months of Incarceration	8	7	7.5	0.81
Study arm assignment				0.39
SAT	14 (28.0%)	16 (36.4%)	30 (31.9%)	
DAART	36 (72.0%)	28 (63.6%)	64 (68.1%)	
Axis I psychiatric diagnoses (DSM-IV)	26 (52.0%)	17 (38.6%)	43 (45.7%)	0.20
Mood disorders	22 (44.0%)	11 (25.0%)	33 (35.1%)	0.05
Anxiety disorders	16 (32.0%)	12 (27.3%)	28 (29.8%)	0.62
Psychotic disorders	7 (14.0%)	6 (13.6%)	13 (13.8%)	0.96
Hazardous Drinking (AUDIT≧8)	13 (26.0%)	15 (34.1%)	28 (29.8%)	0.39
Opioid Craving (≧5)	27 (56.3%)	3 (60.0%)	30 (56.6%)	0.88
Addiction Severity (ASI)				
Alcohol	0.29±0.12	0.27±0.15	0.280±0.137	0.70
Drugs	0.10±0.18	0.27±0.17	0.131±0.187	0.63
Lifetime Cocaine use	48 (96.0%)	41 (93.2%)	89 (94.7%)	0.54
Mean CES-D score	18.36±9.85	18.38±12.32	18.37±11.02	0.70
Major depression using CES-D (score 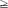 16)	29 (61.7%)	20 (47.6%)	49 (55.1%)	0.18
Mean QIDS score	8.29±4.65	8.44±5.77	8.36±5.18	0.48
Opioid Dependence Score (ODS)	6.24±1.71	6.14±1.58	6.19±1.64	0.86
Social Support Scale	61.49±24.81	66.67±22.26	63.91±23.67	0.75
Trust in Physician	66.86±6.93	68.20±6.93	67.49±6.92	0.68
Health-related quality of life (SF-36)				
Physical Composite Score	46.74±12.38	43.10±13.60	45.04±13.02	-
Mental health composite score	43.15±13.81	44.97±14.03	44.00±13.87	-
Physical function score	74.18±34.44	70.47±32.33	72.45±33.34	0.35
Role-physical score	67.35±43.96	56.98±46.07	62.50±45.01	0.55
Bodily pain score	65.66±35.94	62.03±37.92	63.97±36.72	0.66
General health score	54.18±25.15	51.40±27.70	52.88±26.26	0.02
Vitality score	61.12±23.61	51.05±26.49	56.41±25.37	0.43
Social functioning score	68.11±30.94	66.86±32.15	67.53±31.34	0.35
Role-emotional score	52.38±45.64	66.67±43.64	59.06±45.05	0.12
Mental health score	66.04±22.67	62.70±23.04	64.48±22.78	0.44
Had Methadone prior to incarceration	37 (75.5%)	26 (59.1%)	63 (67.7%)	0.09
Dosing schedule				0.63
Once daily	41 (82.0%)	36 (85.7%)	77 (83.7%)	
Twice daily	9 (18.0%)	6 (14.3%)	15 (16.3%)	
Baseline antiretroviral therapy regimens				0.07
NNRTI+NRTIs	23 (46.0%)	12 (28.6%)	35 (38.0%)	
Boosted PI+NRTIs	22 (44.0%)	22 (52.4%)	44 (47.8%)	
Non-boosted PI+NRTIs	5 (10.0%)	4 (9.5%)	9 (9.8%)	
Others	0	4 (9.5%)	4 (4.4%)	
Viral Load	N = 50	N = 43	N = 93	0.79
HIV-1 RNA<400 copies/mL	36 (72.0%)	32 (74.4%)	68 (73.1%)	
HIV-1 RNA≥400 copies/mL	14 (28.0%)	11 (25.6%)	25 (26.9%)	
Viral Load	N = 50	N = 43	N = 93	0.38
HIV-1 RNA<50 copies/mL	29 (58.0%)	21 (48.8%)	50 (53.8 %)	
HIV-1 RNA≥50 copies/mL	21 (42.0%)	22 (51.2%)	43 (46.2 %)	
Log HIV-1 RNA (among VL>50 copies/mL)	2.41±1.11	2.34±1.04	2.37±1.07	0.59
CD4+ lymphocytes (cells/mL)	375.4±190.9	362.0±261.6	369.1±225.6	0.21

Legend: SAT = Self-administered therapy; DAART = directly administered antiretroviral therapy; ASI = Addiction Severity Index; CES-D = Clinical Epidemiological Survey Depression; QIDS = Quick Inventory Depression Survey; ODS = Opioid dependency Scale; SF = SF-36 QoL = Quality of Life scale; NNRTI = non-nucleoside reverse transcriptase inhibitors; NRTI = nucleoside reverse transcriptase inhibitors; PI = protease inhibitors; VL = viral load.

**Table 2** provides the contribution of various potentially independent effects on the primary outcome, achieving maximal viral suppression (MVS) at 24 weeks. The factors that were found to be statistically significantly associated with achieving MVS at 24 weeks in the univariate analysis were: retention on BPN/NLX for 24 weeks (p = 0.030); male gender (p = 0.047); and having a baseline HIV -1 RNA level of <50 copies/ ML (p<0.001). After inclusion of these covariates in the multivariate regression analysis model separately, retention on BPN/NLX treatment for 24 weeks (regardless of gaps of missing treatment in between), and having a baseline HIV-1 RNA <50 copies/ mL were found to significantly associated with achieving MVS at the end of the 24 weeks among the OD subjects (n = 94), while black race was negatively associated with this primary outcome. The DAART intervention and ‘receiving any buprenorphine or methadone’ were not found to be statistically significantly associated with MVS.

**Table 2:** Correlates of factors associated with maximum viral suppression (HIV-1 RNA<50 copies/ml) among opioid dependent clients at 24 weeks (N = 94).

	Univariate Analysis		Multivariate Model	
	OR (95%CI)	*p* value	AOR (95%CI)	*p* value
**Received any BPN**	1.25 (0.55–2.84)	0.59		
**Received any BPN or methadone**	1.36 (0.59–3.15)	0.47		
**Retained on BPN for 24weeks**	4.32 (1.15–16.2)	0.03*	5.37 (1.15–25.1)	0.03*
**Lifetime Cocaine use**	0.32 (0.03–2.98)	0.32		
**Study Arm**				
** SAT**	referent			
** DAART**	1.56 (0.65–3.74)	0.32		
**Gender**				
** Female**	referent			
** Male**	3.03 (1.01–9.08)	0.05*	4.23 (1.00–18.0)	0.05
**Age**	1.00 (0.94–1.06)	0.99		
**Race/Ethnicity**				
** White**	referent			
** Black**	0.38 (0.11–1.27)	0.12	0.13 (0.03–0.68)	0.02*
** Hispanic**	0.68 (0.20–2.36)	0.55	0.29 (0.06–1.40)	0.12
**Months of incarceration**	1.02 (0.99–1.05)	0.18	1.02 (0.99–1.05)	0.14
**Homeless**	1.09 (0.43–2.73)	0.87		
**Study Site**				
** Hartford**	referent			
** New Haven**	0.78 (0.34–1.83)	0.58		
**DSM-IV psychiatric axis I diagnoses**	0.88 (0.39–2.01)	0.77		
** Mood**	0.83 (0.35–1.96)	0.68		
** Anxiety**	0.98 (0.40–2.40)	0.78		
** Psychotic**	1.22 (0.37–4.04)	0.75		
**Hazardous Drinking**	0.80 (0.33–1.95)	0.62		
**ASI- Alcohol**	0.52 (0.03–10.22)	0.66		
**ASI- Drug**	0.35 (0.04–3.15)	0.35		
**Mean Craving Score (≥5)**	0.88 (0.29–2.62)	0.82		
**Depression (CES-D≥16)**	0.80 (0.34–1.88)	0.61		
**Depression (QIDS>11)**	0.61 (0.25–1.46)	0.27		
**Social Support Scale**	0.99 (0.97–1.01)	0.39		
**Trust in Physician**	1.03 (0.97–1.09)	0.36		
**Quality of Life – Physical Composite Score**	1.00 (0.97–1.04)	0.77		
**Quality of Life – Mental Composite Score**	1.00 (0.97–1.03)	0.99		
**Dosing schedule**	)			
** Once daily**	referent			
** Twice daily**	1.50 (0.47–4.81	0.50		
**Baseline HIV-1 RNA<50 (copies/mL)**	5.91 (2.40–14.6)	<0.001*	10.5 (3.21–34.1)	<0.001*
**AIC**			101.1058	
**BIC**			118.5263	

* binomial variables = 0 is the referent group.

Legend: OR = odds ratio, AOR = adjusted odds ratio; BPN = Buprenorphine; CI = confidence interval; ASI = Addiction Severity Index; Craving = craving for opioids Likert scale 1–10 Dosing Schedule = dosing of cART; SAT = Self-administered Therapy; DAART = Directly Administered Antiretroviral Therapy; QIDS = Quick Inventory Depression Scale; CES-D = Center for Epidemiologic Studies on Depression scale.

**Figure 2:**
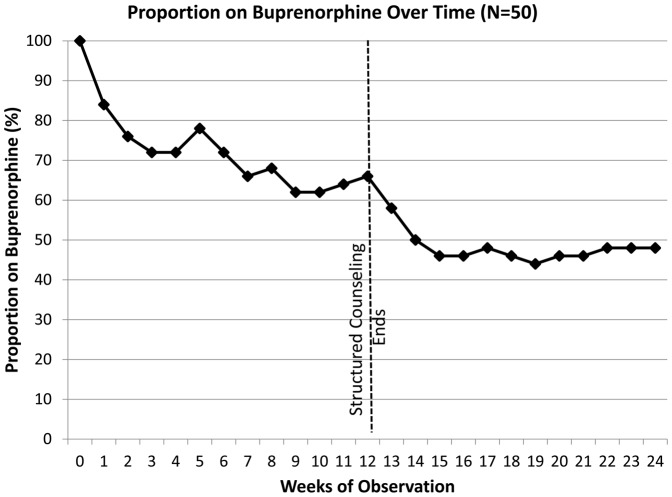
Proportion of Clients on Buprenorphine/Naloxone Over Time. 0: induction; **: N: number of clients receiving buprenorphine.

**Figure 2** shows the percentage of the 50 subjects who were retained on BPN/NLX over the 24 weeks. Forty-two (84%) of these completed the 3-day induction process and nearly half (48%) were retained on BPN/NLX for the entire 24 weeks. As indicated in the figure at the 12-week mark when the mandatory 12-week counseling was completed, there was a significant loss in retention. Reasons for discontinuation of BPN/NLX included reincarceration, hospitalization for issues not related to BPN/NLX, request for opioid pain medications, and in one case, a change to methadone.

**Table 3** depicts the results of the HIV treatment outcomes at baseline compared to 6 months between three naturalistic observation groups: the *Non-BPN group* (n = 44), the *BPN group not retained for 24 weeks* (n = 33) and the *BPN group of subjects who were retained for the full 24 weeks* (n = 17). There was no statistically significant difference for mean CD4 count over the 6 months between the three groups (*p* = 0.38) nor was there for the percentage having a VL <400 copies/mL (p = 0.08). There was, however, a statistically significantly greater percentage of the *BPN group who were retained for 24 weeks* group having MVS over the 6 months when compared to the *Non-BPN* and the *BPN and not Retained for 24 Weeks group* (*p* = 0.01).

**Table 3:** HIV treatment outcomes for Buprenorphine group and Non-Buprenorphine group over 24 weeks.

Outcomes	Time	Non-BPN (N = 44)	BPN But Not Retained 24 Weeks (N = 33 )	BPN And Retained 24 Weeks (N = 17 )	Total Sample Population (N = 94)	*P* value of difference among 3 groups
Mean CD4± SD (cells/mL)	Baseline	370.5± 262.3	384.6± 187.6	360.1± 200.2	373.6± 225.6	
	Week 12	492.1± 386.1	356.1± 237.7	395.3± 241.9	425.2± 316.8	0.38
	Week 24	430.2± 287.7	336.9± 160.3	382.2± 218.6	388.7± 238.9	
HIV-1 RNA<50 copies/ ml, N (%)	Baseline	21 (48.8%)	19 (57.6%)	10 (58.8%)	50 (53.8%)	
	Week 12	20 (45.5%)	14 (42.4%)	13 (76.5%)	47 (50.0%)	0.01
	Week 24	24 (54.6%)	16 (48.5%)	14 (82.4%)	54 (57.5%)	
HIV-1 RNA<400 copies/ ml, N (%)	Baseline	32 (74.4%)	23 (69.7%)	13 (76.5%)	68 (73.1%)	
	Week 12	28 (63.6%)	19 (57.6%)	14 (82.4%)	61 (64.9%)	0.08
	Week 24	31 (70.5%)	22 (66.7%)	15 (88.2%)	68 (72.3%)	

**Figure 3:**
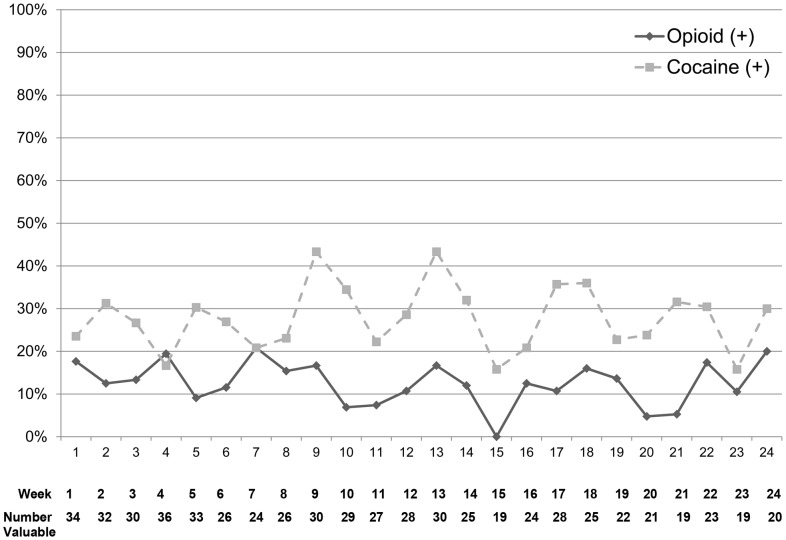
Urine toxicology test results (percent of positive results) among tested clients prescribed buprenorphine/naloxone (N = 50).

**Figure 3** depicts the urine toxicology results that were available for the 50 subjects who began induction with BPN/NLX over the 24 weeks. Eighteen percent had urine toxicology tests positive for opioids at the time of the first day of induction (mean time after release was approximately 7 days), and 20% had urine opioid toxicology tests positive at the end of 24 weeks. Twenty-two percent had urine toxicology tests positive for cocaine at time of induction, and 30% had cocaine positive urine toxicology tests at end of 24 weeks. Only the subjects who were in the BPN-group had weekly urine toxicology results, therefore comparisons with regard to opioid and cocaine use cannot be made between the group that selected BPN and the group that did not.

**Figure 4:**
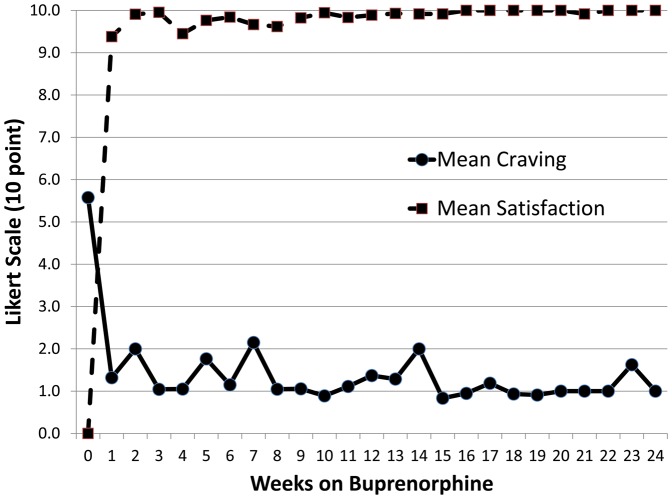
Satisfaction and craving associated with subjects receiving buprenorphine/naloxone.

**Figure 4** depicts the mean opioid craving and satisfaction scores for those receiving BMT over the 24 weeks for the 50 BPN/NLX subjects. The mean opioid craving score was 5.5 at time of baseline induction. This score was reduced to a mean craving score of 1.0 by the end of week 1, and remained consistently there by the 24-week end point. Similarly, satisfaction with BPN/NLX treatment was high with a mean satisfaction score of 9 by the end of the first week of induction rising to a mean of 10 throughout the rest of the 24 weeks of the study.

## Discussion

To our knowledge, this is the largest longitudinal follow-up of HIV-infected prisoners on antiretroviral therapy and meeting pre-incarceration DSM-IV criteria for opioid dependence. The findings from this study have several important implications as described below.

First, being retained on BPN/NLX, along with standardized counseling recommended for HIV-infected patients on BMT, was superior in sustaining maximal viral suppression compared to those not receiving BPN/NLX. This remained true in this naturalistic study where participants could choose their own post-release treatment options, even when controlling for receipt of other OST, including methadone. Unlike our previous pilot BPN/NLX study, we not only examined participants for a longer treatment duration (24 weeks), but also were able to compare outcomes to other opioid dependent participants who did not receive BPN/NLX, including those opting for methadone.

Second, the finding that retention on BMT, along with related counseling and follow-up, was strongly and significantly correlated with MVS confirms findings from other studies where integrating BPN/NLX into HIV clinical care settings is independently associated with viral suppression. [31], [40] [41] Compared to these community-derived samples, this study's subject population had more psychiatric, social and medical co-morbidity, and despite this, retention on BMT was similar. [32] The subjects in this study were highly representative of the criminal justice system with 41% being homeless upon release; 45% having an Axis I DSM-IV mental Illness; 30% meeting criteria for hazardous drinking based on the AUDIT; and 18% having lifetime history of cocaine abuse prior to incarceration. An intervention that can succeed in suppressing HIV viral load in released prisoners who have significant psychosocial comorbidity is highly important. [5] This is particularly salient since most released HIV-infected prisoners engage in high levels of HIV risk taking behaviors [42] and in the absence of other prevention activities (e.g. condom use, risk reduction counseling), cART is an effective approach to reduce HIV transmission. [43].

Third, though the DAART intervention was superior to SAT in the parent trial, it was not independently associated with MVS among these opioid dependent subjects. [32] The multivariate analysis among opioid dependent participants, however, revealed that being retained on BMT, irrespective of DAART randomization, was the mediating factor in sustaining maximal viral suppression. Shorter retention on BPN/NLX and receipt of methadone was not correlated with MVS. This suggests that optimizing the use of OST using BPN/NLX among opioid dependent HIV-infected prisoners, especially with optimal retention on substance abuse treatment, could improve HIV treatment outcomes when released to the community. Although DAART is an effective intervention, OST using BPN/NLX is less costly and should be considered as a potential post-release mechanism to improve HIV treatment outcomes among opioid dependent released prisoners. The extent that the use of other forms of medication-assisted therapy or in treating other substance use disorders including alcohol remains to be determined. [5], [44].

Fourth, craving for opioids is associated with a high rate of opioid relapse among those who have undergone supervised opioid withdrawal. [45] Craving remained high at the time of release in this study, especially for a group who had been incarcerated for many months and after forced abstinence from opioids. Indeed, 20% had already relapsed to opioid use within 7 days after release. Despite their high craving for opioids and relapse to opioid use for some of these subjects, BPN/NLX was successfully started and linked with cART. This highly feasible, acceptable and effective intervention likely assisted in their ability to control the chaos after release by decreasing craving for opioids and allowing them to be adherent to their cART, thereby improving the likelihood of effectively suppressing HIV replication. It is possible that the craving for, and use of cocaine throughout the study may have contributed to poorer treatment outcomes by modifying the effect of BPN/NLX as noted in other studies. [46] Unfortunately urine drug screening was not available for all participants and the effect of continued cocaine use could not be evaluated in the multiple regression analysis.

Integrating BPN/NLX with cART for released OD prisoners has significant public health implications. Suppression of HIV replication will ultimately contribute to decreases in transmission of HIV to the uninfected public. ART alone has been found to reduce spread of HIV infection to serodiscordant couples [4] and among general populations studies in San Francisco [47], Vancouver [48], and in Taiwan [49]. In this study, 53% of the total 94 subjects had MVS at the time of release; and for the BPN group 60% still had MVS at 6 months after release, a truly remarkable feature for a group that tends to lose this benefit within 12 weeks after release. [8], [50] This OST intervention study suggests that treatment using BPN/NLX for opioid dependence among HIV-infected opioid dependent CJS populations who are prescribed cART independently enhances viral suppression, suggesting that BPN/NLX is a crucial component of ‘*enhanced treatment as prevention’*. The extent to which CJS administrators, public health officials and public policy makers adopt BPN/NLX, or any effective MAT for that matter, remains problematic in that such evidence-based interventions are generally not available to CJS populations in the U.S. [30], [51] While these data confirm the benefits of BPN/NLX, future studies should also include evaluating the effect of methadone and extended-release naltrexone on HIV treatment outcomes – the latter being a more recently approved medication for the treatment of alcohol use disorders [15], [17] and opioid dependence. [52] Extended-release naltrexone has the potential for less adherence concerns due to its once monthly injection dosing, as well as having no overdose or diversion concerns due to its opioid antagonist properties compared to the opioid agonist medications.

International guidelines have recently been published that support the use of methadone in HIV-infected patients receiving cART, DAART for released prisoners and the integration of DAART into methadone treatment in community settings. [53] This study provides additional empiric data for the use of BPN/NLX for HIV-infected opioid dependent prisoners transitioning to the community as a means to maximize viral suppression, even in the absence of DAART. The lack of a RCT design and the small sample size does, however, limit the strength of this recommendation.

There are limitations to this study including the relatively small sample size and single study site. Receipt of BPN/NLX was not randomized, therefore potentially introducing selection bias. For example, those selecting BMT may have been more motivated to receive treatment in order to avoid relapse to opioid use. This increased motivation may have influenced adherence to BMT compared to those not selecting BMT. It is clear, however, that this analytic approach represents a real-world sample and reflects the realities of what individuals seek as treatment options. Moreover, it is clear that those selecting BPN/NLX were not just selecting it over methadone because it might provide more freedom in their day to day life, since two thirds of BPN/NLX participants received their BMT daily as observed therapy by virtue of being in the DAART arm. Though future studies should consider deploying a randomized controlled trial (RCT) design to eliminate the possibility of such selection bias, such an approach will not overcome concerns about inclusion of patient preferences since it is unlikely that such a RCT could ever realistically be conducted as placebo-controlled. Additionally, though we know the extent of the counseling provided to the BMT group, the Non-BPN comparison group was not followed as closely and did not include urine toxicology testing. Though counseling has been demonstrated to enhance the benefits of OST, counseling alone would be entirely insufficient to provide significant benefit. [54] Last, there may have been some contributors to retention that were not measured in this study, such as homelessness, mental illness status, social support and motivation. It is unlikely that homelessness contributed differently to outcomes since the two groups did not differ at baseline, nor did their levels of underlying depression or addiction severity. The extent to which these factors contributed to MVS and retention on BPN among the group that retained and not retained on BPN/NLX for the entire 24 weeks is unknown. Despite these limitations, this study not only supports that HIV-infected prisoners transitioning to the community are more likely to accept BPN/NLX treatment to prevent relapse to opioid use than methadone maintenance or even no OST at all, but that retention on BPN/NLX was highly correlated with MVS. This is particularly salient since most had prolonged periods of forced abstinence (mean length of time of 7.5 months) and 63% had been previously treated with methadone maintenance and none had any knowledge about or experience with BPN/NLX upon being screened for this study.

In conclusion, buprenorphine induction upon release for HIV-infected opioid dependent prisoners is highly acceptable and feasible. Retention on buprenorphine, irrespective of other medical or psychiatric comorbidity, evidence-based adherence interventions or use of methadone, was strongly associated with maximal viral suppression. Buprenorphine maintenance treatment, along with concomitant counseling, effectively enhances the benefit of cART as enhanced treatment as prevention for released HIV-infected opioid dependent prisoners and has great potential to improve health from an individual and public perspective.
